# Forecasting Erroneous Neural Machine Translation of Disease Symptoms: Development of Bayesian Probabilistic Classifiers for Cross-Lingual Health Translation

**DOI:** 10.3390/ijerph18189873

**Published:** 2021-09-19

**Authors:** Meng Ji, Wenxiu Xie, Riliu Huang, Xiaobo Qian

**Affiliations:** 1 School of Languages and Cultures, University of Sydney, Sydney 2006, Australia; rhua5035@uni.sydney.edu.au; 2 Department of Computer Science, City University of Hong Kong, Hong Kong 518057, China; Vasiliky@outlook.com; 3 School of Computer Science, South China Normal University, Guangzhou 510631, China; xiaoboqian1221@outlook.com

**Keywords:** machine translation, machine learning, health/medical translation, digital healthcare services, vulnerable people, symptoms translation

## Abstract

Background: Machine translation (MT) technologies have increasing applications in healthcare. Despite their convenience, cost-effectiveness, and constantly improved accuracy, research shows that the use of MT tools in medical or healthcare settings poses risks to vulnerable populations. Objectives: We aimed to develop machine learning classifiers (MNB and RVM) to forecast nuanced yet significant MT errors of clinical symptoms in Chinese neural MT outputs. Methods: We screened human translations of MSD Manuals for information on self-diagnosis of infectious diseases and produced their matching neural MT outputs for subsequent pairwise quality assessment by trained bilingual health researchers. Different feature optimisation and normalisation techniques were used to identify the best feature set. Results: The RVM classifier using optimised, normalised (L_2_ normalisation) semantic features achieved the highest sensitivity, specificity, AUC, and accuracy. MNB achieved similar high performance using the same optimised semantic feature set. The best probability threshold of the best performing RVM classifier was found at 0.6, with a very high positive likelihood ratio (LR+) of 27.82 (95% CI: 3.99, 193.76), and a low negative likelihood ratio (LR−) of 0.19 (95% CI: 0.08, 046), suggesting the high diagnostic utility of our model to predict the probabilities of erroneous MT of disease symptoms to help reverse potential inaccurate self-diagnosis of diseases among vulnerable people without adequate medical knowledge or an ability to ascertain the reliability of MT outputs. Conclusion: Our study demonstrated the viability, flexibility, and efficiency of introducing machine learning models to help promote risk-aware use of MT technologies to achieve optimal, safer digital health outcomes for vulnerable people.

## 1. Introduction

Digital technologies are having increasing applications in healthcare and clinical settings [1,2,3,4,5,6,7,8]. Machine translation (MT) tools are offering rapid, cost-effective solutions to persistent barriers in health communication caused by language issues, compound by other socioeconomic factors such as educational levels, health literacy, cultural backgrounds and so on. The availability, convenience and privacy afforded by online MT tools has enabled better access to health and medical information among vulnerable people and communities. However, the risks and harms of the increasing uptake of these MT tools which are often designed for general purposes [9,10,11,12], in clinical or self-diagnosis settings, are known [13,14,15]. For people with bilingual skills, higher educational or health literacy levels, the effect of these MT tools on their health decision making is largely limited, as people can utilise relevant health knowledge and skills or direct contacts with medical professionals to critically assess the reliability and validity of MT outputs. For vulnerable people, the increasing use of online MT tools without the necessary bilingual skills and medical knowledge can have clinically significant consequences. 

Research has shown that various factors can contribute to erroneous outputs of MT tools when applied in specialised medical or healthcare settings. Contrary to previous models of MT technologies, such as statistical MT or rule-based MT, neural MT tends to outperform in the translation of difficult medical jargons, more complex sentence structures and generate more fluent and natural MT outputs. In our study, we focused on the MT quality issue associated with disease symptoms which are often conveyed in high frequency, polysemous words in a certain language which require higher levels of context-dependent interpretation of their meanings. By contrast with signs, symptoms are the subjective description and assessment of individual health conditions. Important variability in the semantic meanings of symptoms exists between their usage in general language versus specialised domains such as health and medicine. They provide first-hand information from patients to medical professionals in disease diagnosis and confirmation of cases. In health and medical resources developed for educational, promotion purposes, an exact, well-defined use of symptom terms can effectively help people to understand the conditions, progression of their health status. Currently, there is a lack of standardised bilingual vocabularies of symptoms, despite that symptoms are widely used in international guidelines of disease definition and classification, alongside laboratory tests. For example, the inclusion of symptoms in the detection of dengue fever helped increase the specificity of disease screening tools, whereas laboratory tests contributed to higher screening sensitivity [16,17,18,19]. In scenarios of limited healthcare sources, accurate symptom description is more affordable than laboratory tests. 

The translation of underdefined symptom terms poses significant challenges to neural MT systems like Google Translate. Our study aimed to develop effective, affordable research solutions, countermeasures to the MT issue related to symptoms. We developed Bayesian machine learning classifiers to predict the likelihood of MT errors in terms of their treatment of symptoms. The outputs of our models were the probabilities of a certain original English medical text on disease diagnosis which would cause erroneous symptom translation using Google Translate. People and MT users with limited medical knowledge can thus make more informative health decision for themselves and those they care for.

## 2. Materials and Methods

### 2.1. Screening of Original English Source Texts 

To promote the informed use of MT tools to acquire health information through computer-aided translation by vulnerable patients and their caregivers, we developed Bayesian machine learning classifiers to help the public understand the likelihood of inaccurate self-diagnosis based on outputs of online MT applications. The Merk Manual of Diagnosis and Therapy (MSD Manuals) are widely used in health education and family healthcare around the world [20,21]. Its Chinese consumer edition is commissioned to national leading medical professionals of the Chinese Preventive Medicine Association. High-quality human translations of MSD Manuals were used as references to evaluate the quality, reliability of neural machine translation outputs. We screened human, professional translations of MSD Manuals for information on self-diagnosis of infectious diseases and produced their matching neural machine translation outputs for subsequent pairwise quality assessment by trained bilingual health researchers. Pairwise comparison between human and machine translations helped use to identify and verify clinically significant MT errors (kappa coefficient 0.842, 95% CI: 0.762, 0.922) of symptoms which could cause inaccurate self-diagnosis of highly transmissible diseases by consumers of the MSD Manuals. 

### 2.2. Multi-Dimensional Features

Through the observation of the original clinically significant errors in machine translation outputs, the language difficulty, morphological or syntactically complex expressions and the semantic meanings of original English expressions were the main factors contributing to the occurrence of machine translation errors. Thus, the original MSD Manuals were represented by global, high-level, and multi-dimensional features instead of the traditional local lexical features (the frequency/occurrence of words, e.g., bag-of-words). The multi-dimensional features contained both structural and semantic features, which were extracted by two public available English corpus annotation systems. 

### 2.3. Structural Features

The Readability Studio (Oleander Software) was applied to extract a total of 20 morphological and structural features of the original English texts, containing descriptive statistics [22,23,24,25,26]. The structural features consisted of four global features of the original texts of different dimensions: complex sentences (six features), lexical complexity (three features), morphological and orthographic complexity (eight features), and content density (three features). The complex sentence features were average number of sentences per paragraph, number of difficult sentences (more than 22 words), longest sentence, average sentence length, passive voice, and sentences that begin with conjunctions. The lexical complexity features were number of unique words, number of unique long words, and number of unique monosyllabic words. The morphological and orthographical complexity features consisted of number of syllables, average number of characters, average number of syllables, number of monosyllabic words, number of complex (three+ syllable) words, number of unique three+ syllable words, number of long (six+ characters) words, and misspellings. The content density features were number of proper nouns, overused words, and wordy items. 

### 2.4. Semantic Features 

For semantic features, USAS (University of Lancaster Semantic Annotation System) [22,23] was utilized to explore the potential relations between clinically significant symptom errors in MT and the original English words semantic type and expressions. In total, 115 fine-grained semantic features of the original English health texts were extracted and annotated by the USAS semantic system. The extracted 115 features fell into 21 major discourse fields: general and abstract terms (A1–A15, 15 features); the body and the individual (B1–B5, five features); arts and crafts (C1); emotion (E1–E6, six features); food and farming (F1–F4, four features); government and public (G1–G3, three features); architecture, housing and the home (H1–H5, five features); money and commerce in industry (I1–I4, four features); entertainment, sports and games (K1–K6, six features); life and living things (L1–L3, three features); movement, location, travel and transport (M1–M8, eight features); numbers and measurements (N1–N6, six features); substances, materials, objects and equipment (O1–O4, four features); education (P1), language and communication (Q1–Q4, four features); social actions, states and processes (S1–S9, nine features); time (T1–T4, four features); world and environment (W1–W5, five features); psychological actions, states and processes (X1–X9, nine features); science and technology (Y1–Y2, two features); names and grammar (Z0–Z9, Z99, 11 features). These hierarchically arranged semantic types of words gave us a global view of the distribution of semantic meanings of the original English texts on disease diagnosis, which were useful for investigating the importance of the word choice and vocabulary diversity for neural machine translation tools like Google Translate to provide a reliable and accurate translation.

### 2.5. Bayesian Machine Learning Classifiers 

The Bayesian framework-based methods provide probabilistic predictions of given samples and are widely used for assisting decision making in medical research [27,28,29]. Probabilistic learning allows researchers to develop a more intuitive interpretation of uncertainty and make utility assessment interpretable and useful to patients and medical professionals in disease diagnosis. In our study, two Bayesian machine learning classifiers, relevance vector machine (RVM) and multinomial naïve Bayes (MNB), were used to develop to predict MT errors of clinical symptoms in Chinese neural MT outputs. RVM has the identical function as support vector machines (SVM). RVM is known as a sparse classifier, which is not susceptible to the issue of overfitting, as a result of algorithm complexity. RVM suits the development of machine learning classifiers on small data sets like ours because of its enhanced generalization ability [30,31]. MNB is an effective and easy-to-train Bayes theorem-based statistical classification classifier, which works well on categorical text data and highly scalable that is less likely to overfit data [32,33]. 

The collected MSD Manuals (totally 185 samples) were manually annotated as symptom-error-prone (75 samples) and non-symptom-error-prone (110 samples) English health materials. To evaluate the performance of the developed RVM and MNB, the annotated data were randomly split into training data (70%) and testing data (30%) for evaluation. The training data (129 samples) contained 53 English health materials that were symptom-error-prone and 76 English health materials that were non-symptom-error-prone. The testing data (56 samples) contained 22 symptom-error-prone English health materials and 34 non-symptom-error-prone English health materials. We applied both five-fold cross-validation and holdout validation to evaluate the performance of classifiers using five evaluation metrics (accuracy, macro F-score, sensitivity, specificity, and area under the curve, AUC). For five-fold cross-validation, the training data (129 samples) were further randomly split into five subsets. For each fold, the classifier was trained on the selected four subsets and validated on the remaining one. This process was repeated five times during which each subset served as the validation data once. For holdout validation, the classifiers were trained on the training data (129 samples) and validated on the holdout testing data (56 samples).

### 2.6. Feature Optimisation 

The original English texts were represented by a total of 135 multi-dimensional features (20 structural features and 115 semantic features), of which the feature dimension (135) was larger than the number of training data (129). Aiming at discovering a simple and concise yet effective features set to develop a simple model with good generalization ability and lower risk of overfitting, we applied recursive feature elimination (RFE) with support vector machine (SVM) as the base estimator to perform backward feature reduction and remove the features that were unimportant [34]. To obtain a set of features that could produce a stable performance, we performed five-fold cross-validation on training data for recursive feature elimination. The features with higher five-fold cross-validated performance were selected by RFE as the optimised features. 

To explore the relevance between different aspects (morphological and structural complexity only; semantic complexity only; and interaction between morphological and structural complexity and semantic complexity) of original language complexity and symptom-error-prone in machine translations of public health resources, two optimisation techniques were applied to extract the most informative features from the original features. First, the RFE was applied on 20 structural features and 115 semantic features to obtain the best Structural-Optimised Features (TOF) and Semantic-Optimised Features (SOF) separately. Then, we applied RFE to perform joint optimisation on the full 135 multi-dimensional features (Jointly Optimised Features, JOF) to explore the potential interaction and relations between morphological structural features and semantic features. 

Three sets of optimised features were identified by using backward feature selection RFE with two optimisation techniques: First, jointly-optimised features (JOF, 57 features) included the number of difficult sentences (more than 22 words), longest sentence, average sentence length, number of unique words, number of proper nouns, number of monosyllabic words, number of unique monosyllabic words, number of unique 3+ syllable words, number of long (6+ characters) words, number of unique long words, misspellings, overused words, wordy items, passive voice, A1, A2, A3, A4, A5, A6, A7, A9, A10, A13, B1, B2, B3, B4, B5, C1, F1, L1, L2, L3, M2, M6, M7, N1, N3, N5, N6, O1, O2, P1, Q1, Q2, S1, S2, S7, S8, X2, X3, X9, Z5, Z6, Z8, Z99. Second, the structural-optimised features (TOF, 5 features) contained the average number of sentences per paragraph, number of difficult sentences (more than 22 words), number of unique words, number of syllables, and wordy items. Lastly, the semantic-optimised features (SOF, 14 features) contained A2, A3, A4, A6, A7, A13, B1, B2, B3, N5, O1, O2, Z5, and Z99.

Furthermore, to prevent the features with a larger range from dominating the RVM optimisation process, we performed data normalization to scale the data features to improve the model generalization ability [35,36]. MNB, using discrete features (the number of feature occurrences), was not required to perform data normalisation. Two normalization methods were applied in our study: Min-Max normalization (denoted as Min-Max, the data were scaled to a certain range, e.g., [0, 1]) and L_2_–norm normalization (denoted as L_2_, the data samples were scaled individually to the unit norm, i.e., the sum of the squares of the data will always be up to 1).

## 3. Results

**Figure 1:**
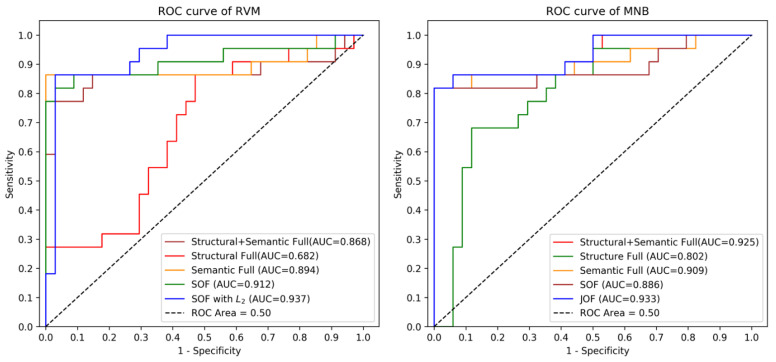
ROC curves of RVM and MNB with different feature sets.

We compared the performance of different methods with different feature sets (structural-optimised features, TOF; semantic-optimised features, SOF; and jointly optimised features JOF) and data normalization techniques (Min-Max and L_2_) with respect to AUC, accuracy, f-score, sensitivity and specificity metrics. The results of five-fold cross-validation (CV) on training data and holdout validation on testing data of different models are shown in Table 1 and Figure 1. For the RVM classifier, the performance of RVM with optimised features always outperformed RVM with non-optimised features (the original full features) on the testing data: using the structural-optimised features, the AUC and specificity of RVM increased from 0.682 and 0.71 (using structural full features) to 0.759 and 0.91, respectively; using semantic-optimised features, the AUC and specificity of RVM increased from 0.894 and 0.91 (using semantic full features) to 0.912 and 0.94, respectively; applying jointly-optimised features, the AUC and sensitivity of RVM increased from 0.77 and 0.868 (using full structural and semantic features) to 0.82 and 0.878, respectively. With data normalisation, the performances of RVM with semantic-optimised features and jointly optimised features were both further improved. The best performing RVM was the one using L_2_ normalised SOF, with an AUC of 0.937, a sensitivity of 0.86 and a specificity of 0.94. For MNB that does not require a data normalization, the best performing model was the one using JOF, with an AUC of 0.933, a sensitivity of 0.82 and a specificity of 0.97. The performance of MNB with optimised features was not less consistently improved on the training data (five-fold CV). 

These results demonstrated that developing a simple yet highly cost-effective model with less features indicative of English health materials prone to symptom errors in neural machine translations was both practicable and applicable. Compared with MNB, RVM with L_2_ normalised SOF had higher AUC, sensitivity and specificity, which was selected as the best performing model for further diagnostic utility assessment and decision making in our study.

To evaluate the suitableness of the Bayesian machine learning classifiers for assessing whether an original English materials would prompt machine translation errors, we compared the performance of RVM and MNB with traditional readability formulas: Flesch Reading Ease Scores (based on average sentence length and average number of syllables per word), Gunning Fog Index (used average sentence length and percentage of hard words) and SMOG Index (used polysyllabic words that had more than three syllables). Applying the readability formulas as binary classifiers, the underlying hypothesis was that there was a positive correlation between the difficulty of English texts and the number of errors in the MT outputs of the original English texts. That is to say, the more difficult the original English health materials were, the more likely the MT systems would produce a machine translation error as defined in our study. Thus, the materials with Flesch Reading Ease Score lower than 60, Gunning Fog Index greater than 12 and SMOG Index greater than 12 were regarded as difficult to read and symptom-error-prone. As shown in Table 1, the performance of readability-formula-based binary classifiers was worse than a random guess (AUC = 0.5), with AUCs of 0.318 (Flesch Reading Ease Scores), 0.277 (Gunning Fog Index) and 0.283 (SMOG Index). This finding suggested that the symptom-error-prone materials were not relevant to the readability and complexity of original English health materials. The easy-to-read materials also had potential to prompt MT systems to produce a clinically significant symptom error. Thus, it is not suitable and reliable to assess whether the machine translation of English source materials would contain symptom errors by utilizing the standard (currently available) readability formulas. The best performing RVM (AUC: 0.937; sensitivity: 0.86; specificity: 0.94) and MNB (AUC: 0.933; sensitivity: 0.82; specificity: 0.97) demonstrated that machine learning methods were more suitable, effective and robust for identifying the symptom-error-prone English health materials on infectious diseases.

Table 2 shows the two-tailed Mann–Whitney U test of RVM with different feature sets on testing data using five evaluation metric results: AUC, accuracy, f-score, sensitivity and specificity. The results showed that the overall performance (considering all five evaluation metrics) of the best performing RVM with L_2_ normalised SOF was statistically significantly improved comparing to RVM using Min-Max normalised TOF (*p*-value: 0.0122, CI: 0.0685 to 0.4043), RVM with JOF (*p*-value: 0.0367, CI: 0.0381 to 0.0715), RVM with structural full feature (*p*-value: 0.0122, CI: 0.1128 to 0.6004), and RVM with structural and semantic features (*p*-value:0.0367, CI: 0.0522 to 0.0367). This result indicates that the semantic features were more informative and effective for identifying the symptom-error-prone English health education materials than morphological and structural features. The machine translation with significant symptom errors was mainly associated with the bilingual vocabularies and expression of symptoms instead of language syntactically complexity (e.g., average number of sentences per paragraph, number of difficult sentences and number of unique words).

## 4. Discussion

### 4.1. Probabilistic Results

Table 3 shows outputs of the readability formula-based binary classifiers and RVM, MNB machine learning classifiers as probabilities of belonging to either symptom-error-prone (SEP), and non-symptom-error-prone (NSEP) English health materials. RVM using L_2_ normalised structural-optimised feature and MNB using structural-optimised feature (5) did not differ significantly between English health materials prone to machine translation errors and those which were not prone to machine translation errors. Outputs of readability formulas-based classifiers and MNB, RVM classifiers using other feature sets differed significantly between two sets of original health materials in English on infectious diseases. The RVM with L_2_ normalised SOF and MNB with SOF had the highest probability means (RVM: 0.802; MNB: 0.818) on SEP English health materials and low probability means (RVM: 0.209; MNB: 0.077) on NSEP English health materials, showing the effectiveness of the semantic-optimised features and the ability of Bayesian machine classifiers for distinguishing between the SEP and NSEP English health materials.

**Figure 2:**
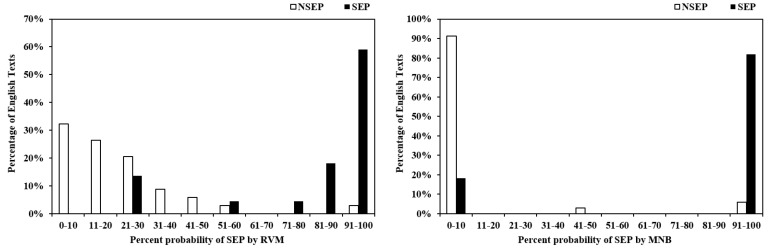
Percentage of symptom-error-prone (SEP) and non-symptom-error-prone (NSEP) English texts assigned by RVM with L_2_ normalised SOF (**left**) and MNB with SOF (**right**) classifier to each 10% probability bin.

Figure 2 shows the histograms that displayed the number of symptom-error-prone (SEP) and non-symptom-error-prone (NSEP) English health materials that fell into each 10% probability bin based on outputs of RVM with L_2_ normalised SOF (left) and MNB with SOF (right). For RVM, 94% of NSEP English health materials were assigned a probability of error-prone < 50% (specificity = 0.94), and 86% of SEP English health materials were assigned a probability of error-prone ≥ 50% (sensitivity = 0.86), showing considerable overlap in outputs between the NSEP and SEP texts. For MNB, 94% of NSEP English health materials were assigned a probability of error-prone < 50% (specificity = 0.94), and 82% of SEP English health materials were assigned a probability of error-prone ≥ 50% (sensitivity = 0.82). Compared to RVM, as shown in Figure 2, the MNB outputs were less overlapped outputs between the NSEP and SEP texts. Thus, RVM was more suitable than MNB for further decision making since it allows the expert to select different thresholds to gain the desired sensitivity and specificity pairings for diagnostic utility based on different criteria. On the other hand, with fewer overlapped outputs, changing the thresholds of MNB will not change the sensitivity and specificity.

### 4.2. Diagnostic Utility 

In Figure 2 (left), nearly 14% of symptom-error-prone MSD manuals were assigned low probabilities of 21–30%. In order to improve the classifier sensitivity, we can adjust the probability thresholds to gain the desired sensitivity and specificity pairings. Table 4 showed that if the probability threshold of the best performing RVM decreased from 0.50 to 0.23, the model sensitivity increased from 0.86 (95% CI: 0.72 to 1.01) to 0.95 (95% CI: 0.87 to 1.04), but the specificity decreased from 0.94 (95% CI: 0.86 to 1.02) to 0.71 (95% CI: 0.55 to 0.86). By contrast, if the probability threshold increased from 0.5 to 0.9, the sensitivity decreased from 0.86 (95% CI: 0.72 to 1.01) to 0.59 (95% CI: 0.39 to 0.80) and the specificity increased from 0.94 (95% CI: 0.86 to 1.02) to 0.97 (95% CI: 0.91 to 1.03). Diagnostic utility (positive likelihood ratio LR+, negative likelihood ratio LR−) was also an effective criterion for evaluation of the assessment tool. The likelihood ratio decided how the prediction changed the probability of certain outputs (positive likelihood ratio was the ratio of sensitivity to false positivity; negative likelihood ratio was the ratio of false negativity and specificity). The assessment tool was regarded as effective and practicable with large positive likelihood ratios and small negative likelihood ratios. Table 4 shows that 0.6 was the best probability threshold for the best performing RVM classifier using the 14 L_2_ normalised semantic-optimised features, including A2 (affect: modify, change, and cause/connected), A3 (being), A4 (classification: generally kinds, groups, examples, particular/ general and detail), A6 (comparing: similar/different, usual/unusual and variety), A7 (definite), A13 (degree), B1 (anatomy and physiology), B2 (health and disease), B3 (medicines and medical treatment), N5 (quantities: entirety, maximum, exceeding and waste), O1 (substances and materials generally: solid, liquid and gas), O2 (objects generally), Z5 (grammatical bin), and Z99 (unmatched). 

## 5. Conclusions

MT technologies offer convenient, cost-effective solutions to existing barriers of access of vulnerable people to healthcare services in multicultural countries. Although the risks and harms of the increasing uptake of MT tools in clinical settings are well documented, limited protective mechanisms or countermeasures have been developed to help alleviate their impact on communities, people who rely on these low-cost technologies to access medical services. Our study demonstrated the viability, flexibility, efficiency of introducing machine learning models to help promote risk-aware use of MT technologies to achieve optimal, safer digital health outcomes for vulnerable people. We found that erroneous neural MT outputs of infectious disease symptoms were associated with a current lack of standardized bilingual vocabularies of symptoms. The interpretation of subjective symptom terms can vary substantially between the general and specialised use of these terms, as well as across individuals: types, severity of pains, ranges and alarming levels of body temperatures, cognitive abilities, consciousness, physical mobility, types of experienced vision problems or disturbances, and malfunction of body parts. These were the symptom terms that were often mistranslated by neural MT tools which could cause misleading self-diagnosis. High-frequency, polysemous symptom words in Chinese require context-dependent approaches to medical translation, for which human translators clearly outperformed neural MT tools. Our research solution to this issue with current neural MT tools when applied in health and medical settings was the development of high-sensitivity machine learning classifiers which could effectively predict the likelihood of erroneous MT outputs in terms of the translation of subjective symptom terms. We believe that the combined use of machine translation and machine learning tools will help add more needed security to online digital health aids and tools and help empower vulnerable communities and people.

## Figures and Tables

**Table 1:** Performance of readability formulas, relevance vector machine (RVM) and multinomial naïve Bayes (MNB) on training and testing data with different features and data normalization methods. CV: cross validation. Bold: to indicate the best model identified.

Methods	Training (5-Fold CV)	Testing				
	AUC Mean (SD)	AUC	Accuracy	F-Score	Sensitivity	Specificity
**Readability Formula Based Binary Classifiers**						
Flesch Reading Ease Scores (60)	/	0.318	0.393	0.28	1	0
Gunning Fog Index (12)	/	0.277	0.321	0.32	0.36	0.29
SMOG Index (12)	/	0.283	0.321	0.32	0.36	0.29
**Machine Learning Classifiers using Full Feature Sets (number of features)**						
Structural Full RVM (20)	0.668 (0.070)	0.682	0.554	0.51	0.32	0.71
Semantic Full RVM (115)	0.801 (0.059)	0.894	0.893	0.89	0.86	0.91
Structural + Semantics Full RVM (135)	0.858 (0.047)	0.868	0.839	0.83	0.77	0.88
Structural Full MNB (20)	0.6957 (0.12)	0.802	0.786	0.77	0.68	0.85
Semantic Full MNB (115)	0.7966 (0.05)	0.909	0.893	0.89	0.82	0.94
Structural + Semantics Full MNB (135)	0.786 (0.058)	0.925	0.911	0.90	0.82	0.97
**Machine Learning Classifiers using Different Optimised Feature Sets (number of features)**						
Structural-optimised (TOF) RVM (5)	0.605 (0.075)	0.759	0.661	0.58	0.27	0.91
Semantic-optimised (SOF) RVM (14)	0.829 (0.042)	0.912	0.893	0.89	0.82	0.94
Jointly-optimised (JOF) RVM (57)	0.846 (0.042)	0.878	0.857	0.85	0.82	0.88
Structural-optimised (TOF) MNB (5)	0.456 (0.118)	0.414	0.554	0.46	0.18	0.79
Semantic-optimised (SOF) MNB (14)	0.839 (0.061)	0.886	0.893	0.89	0.82	0.94
**Jointly-optimised (JOF)** **MNB (57)**	**0.832 (0.061)**	**0.933**	**0.911**	**0.90**	**0.82**	**0.97**
**RVM using Different, Normalized and Optimised Feature Sets (number of features)**						
Structural-optimised (TOF) RVM with Min-Max (5)	0.693 (0.069)	0.691	0.696	0.67	0.5	0.82
Structural-optimised (TOF) RVM with L_2_ (5)	0.345 (0.093)	0.467	0.607	0.38	0	1.0
Semantic-optimised (SOF) RVM with Min-Max (14)	0.847 (0.036)	0.868	0.857	0.84	0.68	0.97
**Semantic-optimised (SOF) RVM with L_2_ (14)**	**0.845 (0.057)**	**0.937**	**0.912**	**0.91**	**0.86**	**0.94**
Jointly-optimised (JOF) RVM with Min-Max (57)	0.787 (0.065)	0.860	0.804	0.80	0.82	0.79
Jointly-optimised (JOF) RVM with L_2_ (57)	0.842 (0.036)	0.947	0.875	0.87	0.86	0.88

**Table 2:** The *p*-value of Mann–Whitney U test (two-tailed) and 95% confidence interval of RVMs using different feature sets (bold values were significant).

RVM Classifier Pair(s)	Asymptotic 95% Confidence Interval		
	Lower	Upper	*p*-Value
SOF with L_2_ vs. Structural + Semantic Full	0.0522	0.0367	**0.0367**
SOF with L_2_ vs. Structural Full	0.1128	0.6004	**0.0122**
SOF with L_2_ vs. Semantic Full	−0.0086	0.0534	0.1412
SOF with L_2_ vs. TOF with Min-Max	0.0685	0.4043	**0.0122**
SOF with L_2_ vs. JOF	0.0381	0.0715	**0.0367**
SOF with L_2_ vs. JOF with L_2_	−0.0321	0.0829	0.4633
SOF with L_2_ vs. SOF	−0.0073	0.0489	0.5284
TOF with Min-Max vs. Structural Full	−0.2146	0.2934	0.8345
JOF with L_2_ vs. Structural + Semantic Full	−0.0219	0.1199	0.1161

**Table 3:** Comparison of readability formula and MLC (RVM, MNB) output between symptom-error-prone (SEP) and non-symptom error-prone (NSEP) English texts (machine learning classifier outputs were assigned probabilities). Bold: bold values were significant.

Techniques	NSEP English Health Materials	SEP English Health Materials	*p* *
	Mean Probability, SD (*n* = 34)	Mean Probability, SD (*n* = 22)	
Flesch Reading Ease Scores (60)	41.088, 9.333	47.591, 10.680	0.0229
Gunning Fog Index (12)	12.774, 1.762	11.232, 1.965	0.0053
SMOG Index (12)	12.694, 1.294	11.582, 1.278	0.0067
Structural Full RVM (20)	0.344, 0.169	0.479, 0.216	0.0230
Semantic Full RVM (115)	0.202, 0.147	0.769, 0.312	<0.00001
Structural-optimised (TOF) RVM (5)	0.370, 0.139	0.451, 0.108	0.0012
Semantic-optimised (SOF) RVM (14)	0.192, 0.171	0.780, 0.290	<0.00001
Structural Full MNB (20)	0.167, 0.321	0.626, 0.431	0.0002
Semantic Full MNB (115)	0.066, 0.239	0.818, 0.394	<0.00001
Structural-optimised (TOF) MNB (5)	0.401, 0.148	0.358, 0.139	0.2867
**Semantic-optimised (SOF) MNB (14)**	**0.077, 0.241**	**0.818, 0.394**	**<0.00001**
Structural + Semantics Full RVM (135)	0.220, 0.186	0.759, 0.324	<0.00001
Structural + Semantics Full MNB (135)	0.042, 0.178	0.817, 0.394	<0.00001
Jointly-optimised (JOF) RVM (57)	0.201, 0.177	0.776, 0.323	<0.00001
Jointly-optimised (JOF) MNB (57)	0.034, 0.147	0.815, 0.393	<0.00001
Structural-optimised (TOF) RVM with Min-Max (5)	0.353, 0.211	0.511, 0.247	0.0168
Structural-optimised (TOF) RVM with L_2_ (5)	0.432, 0.0003	0.432, 0.0003	0.6811
Semantic-optimised (SOF) RVM with Min-Max (14)	0.241, 0.127	0.715, 0.336	<0.00001
**Semantic-optimised (SOF) RVM with L_2_ (14)**	**0.209, 0.180**	**0.802, 0.250**	**<0.00001**
Jointly-optimised (JOF) RVM with Min-Max (57)	0.243, 0.239	0.744, 0.339	<0.00001
Jointly-optimised (JOF) RVM with L_2_ (57)	0.224, 0.193	0.789, 0.232	<0.00001

* *p* values of Mann–Whitney U test.

**Table 4:** Probability thresholds of the best performing RVM using L_2_ normalised semantic-optimised features. Bold: bold values were significant.

Thresholds	Sensitivity (95% CI)	Specificity (95% CI)	Positive Likelihood Ratio (95% CI)	Negative Likelihood Ratio (95% CI)
0.23	0.95 (0.87, 1.04)	0.71 (0.55, 0.86)	3.25 (1.91, 5.51)	0.06 (0.01, 0.44)
0.25	0.91 (0.79, 1.03)	0.74 (0.59, 0.88)	3.43 (1.93, 6.11)	0.12 (0.03, 0.47)
0.40	0.86 (0.72, 1.01)	0.88 (0.77, 0.99)	7.34 (2.88, 18.71)	0.16 (0.05, 0.45)
0.50	0.86 (0.72, 1.01)	0.94 (0.86, 1.02)	14.68 (3.79, 56.90)	0.15 (0.05, 0.43)
**0.60**	**0.82 (0.66, 0.98)**	**0.97 (0.91, 1.00)**	**27.82 (3.99, 193.76)**	**0.19 (0.08, 0.46)**
0.80	0.77 (0.60, 0.95)	0.97 (0.91,1.03)	26.27 (3.76, 183.59)	0.23 (0.11, 0.51)
0.90	0.59 (0.39, 0.80)	0.97 (0.91,1.03)	20.09 (2.82, 142.92)	0.42 (0.25, 0.70)

## Data Availability

Not approval.
